# How much liver does a person need?

**DOI:** 10.1002/ccr3.2552

**Published:** 2019-11-18

**Authors:** Igor Alexander Harsch

**Affiliations:** ^1^ Department of Internal Medicine II Division of Endocrinology and Metabolism Thuringia Clinic “Georgius Agricola” Saalfeld Germany

**Keywords:** abdominal compression, liver cyst

## Abstract

In patients who had no medical care for years, it is wise to expect the unexpected. Here, a gigantic liver cyst compressed abdominal organs, vessels, and the gut. The patient is thus far doing well in a nursing home, exemplifying the low amount of residual liver tissue necessary for survival.

## Graphical abstract

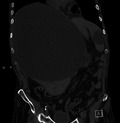

## 1 CASE REPORT

Liver cysts are not an uncommon finding. In elderly patients without medical care for long time, they may reach critical diameters combined with a compression of abdominal organs, vessels, and the gut. Other potential problems are rupture or bleeding of the cyst(s) or an infection of the cyst(s).

**Figure 1:**
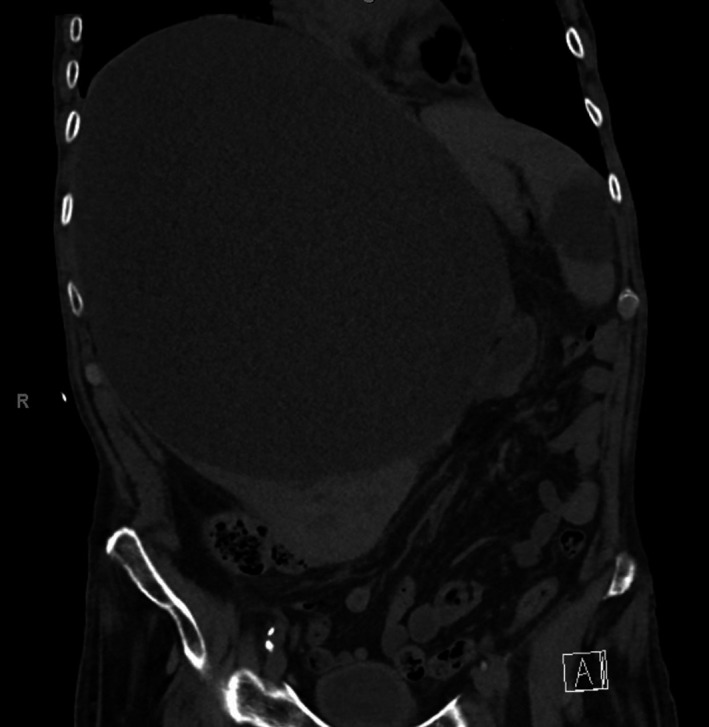
Computed tomography of the abdomen, coronal section: Huge liver cyst with displacement of the upper abdominal organs, gastrointestinal tract, and intra‐abdominal vessels

An 82‐year‐old patient suffered a syncope and was found bedraggled at home in February 2019. The patient had hardly any social contacts in recent years and had not been to a doctor for a long time. He had anemia, but normal serum transaminase and blood coagulation levels. Only cholinesterase was reduced with 77 μkat/L (normal range: 89‐215). An ultrasonography revealed a huge liver cyst, the computed tomography scan of the abdomen revealed the cystic lesion sized 25 × 19 cm, filling almost the entire right lobe of the liver (Figure 1). It caused a diaphragmatic elevation on the right and a compression of abdominal organs. There was no hint of malignancy in other organs. The patient declined a puncture to achieve at least a transient volume relief. Surgery was not possible in his general condition.

Liver cysts are frequent: In an ultrasound screening by Larssen et al^1^ of 1541 patients, cysts are described in 11.3%. In more than 90% of cases, they were 3 cm in diameter or smaller, the incidence increasing with age. Usually, no therapy is required. In liver surgery, a residual tissue mass of 20%‐30% is considered sufficient for functional maintenance in healthy people.^2^ Unfortunately, self‐care by the patient was no longer possible; he was transferred to a nursing home. He is doing well there, and the case described here exemplifies how little liver remnant parenchyma is needed to survive.

## CONFLICT OF INTEREST

There are no conflicts of interest.

## AUTHOR CONTRIBUTIONS

IAH wrote the article and has accountability for all aspects of the work.

## ETHICAL APPROVAL

The patient gave written consent to report his case and the imaging.
